# Use of platelet rich plasma to treat plantar fasciitis: design of a multi centre randomized controlled trial

**DOI:** 10.1186/1471-2474-11-69

**Published:** 2010-04-14

**Authors:** Joost C Peerbooms, Wilbert van Laar, Frank Faber, Hans M Schuller, Henk van der Hoeven, Taco Gosens

**Affiliations:** 1 Department of Orthopaedics, Albert Schweitzer Ziekenhuis, Dordrecht, The Netherlands; 2 Department of Orthopaedics, HAGA Ziekenhuis Den Haag, The Netherlands; 3 Department of Orthopaedics, Diaconessenhuis Leiden, The Netherlands; 4 Department of Orthopaedics, St. Antonius Ziekenhuis Nieuwegein, The Netherlands; 5 Department of Orthopaedics, Elisabeth Ziekenhuis Tilburg, The Netherlands

## Abstract

**Background:**

If conservative treatment for chronic plantar fasciitis fails, often a corticosteroid injection is given. Corticosteroid injection gives temporarily pain reduction, but no healing. Blood platelets initiate the natural healing rate. GPS^® ^gives an eightfold concentrate platelets of patients own blood. Injection of these platelets in the attachment of the fascia to the os calcis might induce a healing rate.

**Methods and design:**

A randomized controlled multi centre trial will be performed. The study population consists of 120 patients of 18 years and older. Patients with chronic plantar fasciitis will be allocated randomly to have a steroid injection or an autologous platelet concentrate injections. Data will be collected before the procedure, 4,8,12,26 weeks and 1 year after the procedure.

The main outcome measures of this study are pain and function measured with questionnaires.

**Conclusion:**

Recent literature show positive effects for the treatment of tendinosis with autologous platelet injections. The forthcoming trial will compare treatment for chronic plantar fasciitis with a steroid injection versus an autologous platelet injection. Our results will be published as soon as they become available.

**Trial Registration:**

Trial registration number: http://www.clinicaltrials.gov NCT00758641.

## Background

Chronic plantar fasciitis is the most common cause of foot complaints in the United States, making up 11 to 15% of the foot symptoms requiring professional care among adults [1,2]. The incidence of plantar fasciitis peaks in people between the ages of 40 to 60 years with no bias towards either sex [3].

The underlying condition that causes plantar fasciitis is a degenerative tissue condition that occurs near the site of origin of the plantar fascia at the medial tuberosity of the calcaneous [4]. In acute cases, plantar fasciitis is characterized by classical signs of inflammation including pain, swelling and loss of function. For more chronic conditions, however, inflammation is not the underlying tissue disruption. In fact, histology of chronic cases has shown no signs of inflammatory cell invasion into the affected area [5]. The tissue instead is characterized histologically by infiltration with macrophages, lymphocytes, and plasma cells; tissue destruction; and repair involving immature vascularization and fibrosis [5]. The normal fascia tissue is replaced by an angiofibroblastic hyperplastic tissue which spreads itself throughout the surrounding tissue creating a self-perpetuating cycle of degeneration [5]. Numerous methods have been advocated for treating plantar fasciitis, including rest, nonsteroidal anti-inflammatory medication, night splints, foot orthosis, stretching protocols and extracorporeal shock wave therapy. Steroid injections are a popular method of treating the condition but only seem to be useful in the short term and only to a small degree [6]. Other various types of surgical procedures have also been recommended [2,7-11]. The use of corticosteroids is particularly troubling as several studies have linked plantar fascia rupture to repeated local injections of a corticosteroid [2,11-13]. When neither rest and neither activity restriction nor conservative treatments result in a satisfactory outcome, the patient is often interested in treatment options other than surgery.

In an animal model the addition of growth factors to the ruptured tendon has been shown to increase the healing of the tendon [14,15]. In humans it has been shown that the injection of whole blood into the tendon decreases the pain [16].

PRP is promoted as an ideal autologous biological blood-derived product, which can be exogenously applied to various tissues where it releases high concentrations of platelet derived growth factors that enhance wound healing, bone healing and also tendon healing. In addition PRP possesses antimicrobial properties that may contribute to the prevention of infections [17]. When platelets become activated, growth factors are released and initiate the body's natural healing response.

We will evaluating the effects of PRP injections on "self-reported function and pain" in a double-blind randomized trial.

## Methods and design

### Study design

Randomization will be performed after patients are deemed eligible and have provided informed consent. Patients will be randomly allocated to the concentrated autologous platelet group (PRP group) or to the corticosteroid group (control group). A computer using block randomisation of 10 patients will be used to create a randomization schedule. Treatment assignments (placed in sequentially numbered opaque envelopes) will be assigned by the trial managers (WL, HS, HH, TG) who will also arrange the facilities needed for the procedure.

All patients with a plantar fasciitis who are admitted to one of the participating hospitals and meet the inclusion criteria are asked to join the study.

Plantar fasciitis was defined as pain at the point of the fascia plantaris origin at direct palpation. All affected patients were screened with X-ray of the calcaneus for bony abnormalities and to difentiate for subtalar arthritis. Sonography and MRI were not used standardly.

The Medical Ethical Committee of The Netherlands approved the study design, procedures and informed consent.

Trial registration number: http://www.clinicaltrials.gov NCT00758641.

### Study population

The study will be conducted at the Orthopaedic Departments of the HAGA Ziekenhuis Den Haag, St. Antonius Ziekenhuis Nieuwegein, Diaconessenhuis Leiden and St. Elisabeth Ziekenhuis Tilburg between November 2008 and December 2010. Authors JP and TG will be responsible for the data and safety monitoring. Inclusion criteria: patients aged > 18 years, with plantar fasciitis (6-12 months duration), who failed conservative treatment are included. They have to be able to understand the informed consent and have a VAS pain score in the morning by first steps higher as 5 (0-10 scale).

Patients will be excluded from the study when they received local steroid injections within 6 months, physical/occupational therapies within 4 weeks, or non-steroidal anti-inflammatory within 1 week prior to randomization. Also patients will be excluded with the inability to fulfil follow-up criteria, significant cardiovascular, renal or hepatic disease, pregnancy, (local) malignancy, history of amenia (hemoglobin < 5.0), previous surgery for plantar fasciitis, active bilateral plantar fasciitis, diagnosis of vascular insufficiency or neuropathy related to heel pain, hypothyroidism and diabetics.

### Intervention

#### Platelet Concentrate Preparation

Fifty-five milliliters whole blood is collected from the uninvolved arm into a 60-mL syringe that contained 5 mL sodium citrate. A peripheral complete blood count is also collected at the time of the initial blood draw. The blood is then prepared according to the GPS System instructions (Cell Factor Technologies, Warsaw, Ind). This device is a desktop-size centrifuge with disposable cylinders for the blood approximately 0.05cc Platelet concentrate is obtained for each patient.

Autologous platelet concentrate contains concentrated white blood cells and platelets that are suspended in plasma. Since an acidic anticoagulant is introduced to the whole blood used to produce the platelet concentrate, the platelet concentrate must be buffered to increase the pH to normal physiologic levels. This is accomplished with 8.4% sodium bicarbonate solution added at a ratio 0.05cc of sodium bicarbonate solution to 1 cc of platelet concentrate.

The resulting buffered platelet concentrate contains approximately a 6 to 8 times concentration of platelets compared to baseline whole blood. No activating agent is used. The total time from blood draw to injection in the patients is about 30 minutes. No specialized equipment, other than the GPS machine, is required.

#### Injection Technique

Initially, bupivacaine is infiltrated into the skin and subcutaneous tissue of both groups as a local field block. Approximately 0.05cc is also injected directly into the area of maximum tenderness. Then, either 5 to 6 cc platelet concentrate or 5 to 6cc corticosteroid is injected using a 22-g needle into the plantar fasciitis using a peppering technique. This technique involved a single skin portal and then 5 penetrations of the fascia.

#### Post-procedure Protocol

Immediately after the injection, the patient is kept in sitting position without moving the foot for 15 minutes. Patients will go to the physiotherapist to obtain stretching exercises. Patients are sent home with instructions to limit their use of the feet for approximately 48 hours and use hydrocodone or acetaminophen for pain. The use of nonsteroidal medication is prohibited. After 48 hours, patients are given a standardized stretching protocol to follow for 2 weeks. A formal strengthening program is initiated after this stretching. At 4 weeks after the procedure, patients are allowed to proceed with normal sporting or recreational activities as tolerated. Any type of foot orthoses will not be allowed.

#### The corticosteroid

The type of steroid that is used during the study is kenacort 40 mg/ml triamcinolon acetonide.

### Study parameters/endpoints

#### Main study pain

Pain will be measured using a visual analogous scale at all time points. The VAS score of the Foot Function Index will be used.

The score records the patient's reported pain using a scale of 0-10, where 0 is pain-free and 10 is the worst pain imaginable. The scale will be a 10 centimeter line beginning with 0 and ending with 10, the score will be marked at the point on the line that corresponds with the patient response.

Each patient randomly assigned to a treatment and with at least one non-missing pre- and post-baseline measurement, will be classified at each visit, as either a treatment success or failure. Patients with a score reduction of 25% compared to baseline, did not require pain medication beyond the protocol defined allowable period, and did not require escape therapy will be considered a treatment success.

The absolute change from baseline to endpoint means the baseline value is subtracted from the endpoint value. Percent change is defined as the absolute change multiplied by 100 divided by the baseline value. For patients whose pain improves, these values will be less than zero.

The treatment is defined as successful if the pain reduction after 6 months is over 25%. If the patient is lost to follow-up, the last measurement will be carried forward. If the patient has obtained a different treatment, the subject is classified as unsuccessful.

#### Function and quality of life

The function and satisfaction will be measured using the AOFAS foot questionnaire [18,19], the Foot Function Index (FFI) [20-22] and WHOQOL quality of life questionnaire [23,24] questionnaires.

#### Follow up

All patients will be followed up at 4, 8, 12, 26 and 52 weeks. All patients will complete AOFAS and VAS scores at all follow up moments

#### Determination of sample size

This study has two main parameters: number of patients with 25% pain reduction after 6 months and the average pain reduction;

Corticosteroids and stretching have a recurrence of pain after 6 months [2,25]. Previous studies with platelet rich plasma by tendonitis showed that the platelet rich plasma group has no recurrence. These results showed 63 percent success rate (VAS reduction over 25 percent after 6 months) for the platelet rich plasma group compared to 27 percent success rate for the corticosteroid group. If these percentages are comparable for plantar fasciitis 38 patients are needed in all groups to achieve a 25% pain reduction (alfa 0,05 and power 0,9).

Conservative treatment with taping or stretching shows a 30 ± 24 points decrease in VAS pain at walking [26,27]. Corticosteroid injections have shown to have comparable results [2,25]. 25% increase in pain reduction is seen as clinical relevant. To prove a difference in pain reduction of 15 ± 24 points and a α of 0,05 and a power of 0,9, 55 patients are needed in each group.

#### Needed number of patients

**Figure 1:**
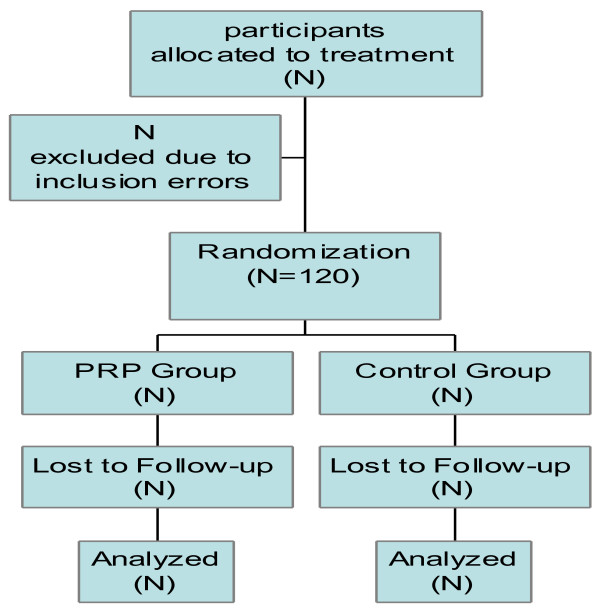
**Flow chart**.

To be able to measure a difference in average pain reduction, the largest number of patient is needed 55 patients in both groups. To compensate for possible lost to follow-up, 60 patients will be included in each group (Figure 1).

#### Statistical analysis

This is a double blind randomized and prospective study. Patients will be randomized in equal proportions to either autologous platelet concentrate or corticosteroid injections. The primary endpoint of the trial is treatment success at 6 months. The other measurements are to measure the speed of recovery and the re-occurrence of pain.

Statistical analyses will test the null hypotheses of no differences between patients treated with autologous platelet concentrate and those treated with corticosteroid in the proportion of the protocol defined, successfully treated patients.

All tests of treatment effect will be made using a one-sided alpha level of 0.025, unless otherwise specified. Tests for the interaction between treatment and a blocking factor will be made at the alpha level of 0.10. All p-values will be rounded then quoted to 3 decimal places, unless the true p-value is less than 0.001 whereby the notation (p < .001) will be used or if the true p-value is greater than 0.999, in which case the notation (p > .999) will be used. Confidence intervals, where indicated for the difference between the two treatment groups, will be calculated at the 95% level. All data will be analyzed by a blinded researcher.

## Discussion

This randomized study is designed to test the use of concentrated autologous platelets in patients with plantar fasciitis. Plantar fasciitis is a common problem with many available treatment methods. When conservative treatment results in a non-satisfactory outcome, the patient is often interested in treatment options other than surgery. Steroid injections are a popular method of treating the condition but only seem to be useful in the short term and only to a small degree [6]. Treatment with corticosteroids has a high frequency of relapse and recurrence, probably because intra fascial injection may lead to permanent adverse changes within the structure of the fascia and because patients tend to overuse the foot after injection as a result of direct pain relief [28].

In a recent study of Peerbooms et al. [29] a positive effect of injection of PRP in the common extension origin for lateral epicondylitis was seen. This report describes the first comparison of an autologous platelet concentrate with corticosteroid injection as a treatment for lateral epicondylitis in patients who have failed non-operative treatment. It demonstrates that a single injection of concentrated autologous platelets improves pain and function more than corticosteroid injection. These improvements were sustained over time with no reported complications [29].

The injection of platelet-rich-plasma (PRP) into the effected tissue addresses the healing stages necessary to reverse the degenerative process which are going on in the base of the plantar fascia. The individual cytokines present in the platelet α-granules have been shown to enhance fibroblast migration and proliferation, up-regulate vascularization, and increases collagen deposition in a variety of in vitro and in vivo settings [30]. The cytokines present in platelet α-granules have been shown to affect the healing stages necessary to reverse a chronic plantar fasciitis condition [30]. Additionally, many of these cytokines have been seen to work in a dose dependent manner [30].

The treatment of tendinosis with an injection of concentrated autologous platelets may be a nonoperative alternative. Utilising the Recover system the patient's own platelets can be collected into a highly concentrated formula. This treatment concept directly addresses the existing condition and should prove to be a superior alternative to current conservative treatments for chronic plantar fasciitis.

We postulate that the concentrated growth factors work in a synergetic manner to initiate a tendon healing response. This hypothesis is supported by in vitro research in the literature. Transforming growth factor β1 is shown to significantly increase type I collagen production by tendon sheath fibroblasts. This same mechanism is likely to be active in chronic plantar fasciitis [31].

## Competing interests

The authors declare that they have no competing interests.

## Authors' contributions

TG originated the idea for the study, led its design and will supervise the project. TG and JP will coordinate the trial and are responsible for data acquisition. JP, WL, FF, HS and HH provided extra protocol information in order to expand the original study design from single-centre to multi-centre. TG, JP, WL, FF, HS, and HH are responsible for data collection at their respective locations. All authors have read and corrected draft versions of the manuscript and have approved the final manuscript.

## Pre-publication history

The pre-publication history for this paper can be accessed here:

http://www.biomedcentral.com/1471-2474/11/69/prepub
